# Trajectory of Long-Term Outcome in Severe Pediatric Diffuse Axonal Injury: An Exploratory Study

**DOI:** 10.3389/fneur.2021.704576

**Published:** 2021-09-14

**Authors:** Shih-Shan Lang, Todd Kilbaugh, Stuart Friess, Susan Sotardi, Chong Tae Kim, Vanessa Mazandi, Bingqing Zhang, Phillip B. Storm, Gregory G. Heuer, Alexander Tucker, Steve B. Ampah, Heather Griffis, Ramesh Raghupathi, Jimmy W. Huh

**Affiliations:** ^1^Division of Neurosurgery, Department of Neurosurgery, Children's Hospital of Philadelphia, University of Pennsylvania Perelman School of Medicine, Philadelphia, PA, United States; ^2^Department of Anesthesiology and Critical Care Medicine, Children's Hospital of Philadelphia, University of Pennsylvania Perelman School of Medicine, Philadelphia, PA, United States; ^3^Department of Pediatrics, St. Louis Children's Hospital, Washington University in St. Louis School of Medicine, St. Louis, MO, United States; ^4^Department of Radiology and Pediatrics, Children's Hospital of Philadelphia, University of Pennsylvania Perelman School of Medicine, Philadelphia, PA, United States; ^5^Department of Physical Medicine and Rehabilitation and Pediatrics, Children's Hospital of Philadelphia, University of Pennsylvania Perelman School of Medicine, Philadelphia, PA, United States; ^6^Data Science and Biostatistics Unit, Department of Biomedical and Health Informatics, Children's Hospital of Philadelphia, Philadelphia, PA, United States; ^7^Department of Neurobiology and Anatomy, Drexel University College of Medicine, Philadelphia, PA, United States

**Keywords:** diffuse axonal injury (DAI), outcome, fever, intracranial hypertension (IH), traumatic brain injury, pediatric

## Abstract

**Introduction:** Pediatric severe traumatic brain injury (TBI) is one of the leading causes of disability and death. One of the classic pathoanatomic brain injury lesions following severe pediatric TBI is diffuse (multifocal) axonal injury (DAI). In this single institution study, our overarching goal was to describe the clinical characteristics and long-term outcome trajectory of severe pediatric TBI patients with DAI.

**Methods:** Pediatric patients (<18 years of age) with severe TBI who had DAI were retrospectively reviewed. We evaluated the effect of age, sex, Glasgow Coma Scale (GCS) score, early fever ≥ 38.5°C during the first day post-injury, the extent of ICP-directed therapy needed with the Pediatric Intensity Level of Therapy (PILOT) score, and MRI within the first week following trauma and analyzed their association with outcome using the Glasgow Outcome Score—Extended (GOS-E) scale at discharge, 6 months, 1, 5, and 10 years following injury.

**Results:** Fifty-six pediatric patients with severe traumatic DAI were analyzed. The majority of the patients were >5 years of age and male. There were 2 mortalities. At discharge, 56% (30/54) of the surviving patients had unfavorable outcome. Sixty five percent (35/54) of surviving children were followed up to 10 years post-injury, and 71% (25/35) of them made a favorable recovery. Early fever and extensive DAI on MRI were associated with worse long-term outcomes.

**Conclusion:** We describe the long-term trajectory outcome of severe pediatric TBI patients with pure DAI. While this was a single institution study with a small sample size, the majority of the children survived. Over one-third of our surviving children were lost to follow-up. Of the surviving children who had follow-up for 10 years after injury, the majority of these children made a favorable recovery.

## Introduction

Severe pediatric traumatic brain injury (TBI) remains one of the leading causes of long-term morbidity and mortality (1–6). While accidental TBI-related deaths in children have decreased overall in the past two decades, survivors are often afflicted with long-term sequelae such as cognitive, psychosocial, and physical disabilities, employment problems and a lower quality of life (6–15). It is becoming increasingly recognized that TBI sustained in childhood is a lifelong chronic condition. In adults, TBI has been recognized as one of the acquired diseases that leads to chronic health problems termed “chronic brain injury” (16).

One of the foundations of acute post-traumatic neurocritical care in severe pediatric TBI [defined as a Glasgow Coma Scale (GCS) of ≤ 8] is to prevent or treat intracranial hypertension as elevated intracranial pressure is an important early pathophysiologic risk factor that has been associated with worse outcomes (17–19). Another potential pathophysiologic risk factor following severe pediatric TBI is early post-traumatic fever or hyperthermia. A few studies have demonstrated that early fever following severe TBI in children was associated with worse hospital discharge outcomes (20, 21), but to our knowledge, no long-term outcome studies have been reported. Age at the time of injury is another factor that may affect outcome following severe pediatric TBI with younger age being associated with worse outcomes in some studies (22–24). Sex differences and the effects on outcome following TBI in children is an area of increasing clinical investigation with conflicting evidence (25–29).

One of the challenges in understanding how pediatric TBI affects outcome is that even within the same level of initial injury severity such as “severe TBI” based on the initial GCS classification, there may be great heterogeneity in the type of lesion(s) present in each individual child (e.g., epidural hematoma vs. subdural hematoma vs. contusion vs. diffuse axonal injury vs. diffuse cerebral edema or a combination) (30, 31). While clearly the severity of initial injury is important, there has been an impetus in the TBI community to classify a particular, specific pathoanatomic brain injury pattern in order to better understand the early pathophysiologic sequelae of that particular injury pattern so that future clinical TBI therapeutic trials can be targeted to a particular TBI subtype (30).

One of the classic pathoanatomic brain injury lesions following severe pediatric TBI is diffuse (multifocal) axonal injury (DAI). Rotational and rapid acceleration-deceleration forces to the brain can lead to widespread axonal white matter shearing and tearing (32). Children are thought to be particularly at risk to these types of shearing injuries due to the relatively decreased myelin content and higher water content in the pediatric brain (33, 34). Following DAI, as the loss of white matter integrity causes neural network connectivity disruptions, acute and long-term neurobehavioral outcomes can be negatively affected (35, 36). However, functional outcome after DAI is difficult to predict as some children have profound disability while others make a better recovery (37–42). A multitude of sophisticated neuroimaging studies have been performed to correlate pediatric DAI and outcome (41, 43–47).

In this single institution study, our overarching goal was to describe the clinical characteristics and long-term outcome trajectory of severe pediatric TBI patients with DAI.

## Methods

### Cohort Selection

This retrospective observational study was conducted at a quaternary children's hospital over a 17-year time period (January 1, 2002–December 31, 2019). The protocol was approved by the Committee for the Protection of Human Subjects Institutional Review Board (IRB). Inclusion criteria included age < 18 years of age at the time of injury, no past medical history, accidental severe TBI with Glasgow Coma Scale (GCS) ≤ 8 score, admission CT concerning for DAI with microhemorrhages in the white matter tracts (48, 49) without a focal mass lesion, and the presence of an intraparenchymal ICP monitoring device. Patients received no other neurosurgical procedures except the intraparenchymal ICP monitor. The ICP was continuously monitored. All of our patients had reactive pupils on admission. Exclusion criteria included penetrating or abusive head trauma, fixed and dilated pupils on arrival, extracranial injuries, anemia, thrombocytopenia or coagulopathy for age, known infection, and pre-existing neurological, psychiatric, developmental disorder, or other medical conditions.

### Variables of Interest

Age and sex were recorded upon admission to the Pediatric Intensive Care Unit (PICU). Early fever was defined as T ≥ 38.5°C during the initial 1st day post-injury (rectal). Routine initial pediatric neurocritical care management included the following: supine position with the head of bed (HOB) elevated at 30 degrees; intubation and ventilation to normocarbia (arterial CO2 35–39 mm Hg); adequate oxygenation (pulse oximetry saturation of 92–98%); analgesia/sedation with fentanyl and/or midazolam; neuromuscular blockade with vecuronium as needed; and phenylephrine or norepinephrine as needed to increase mean arterial pressure (MAP) to maintain minimum age dependent CPP at 40–65 mm Hg. Treatment for intracranial hypertension (defined as ICP ≥ 20 mm Hg for at least 5 min) included a progression of Tier 1 therapies (sedation, analgesia, hyperosmolar therapy, neuromuscular blockade) if needed. Refractory intracranial hypertension required a progression to Tier 2 therapies (hyperventilation, barbiturates, induced moderate hypothermia) if needed. Our treatment protocol was consistent with the “Guidelines for the acute medical management of severe traumatic brain injury in infants, children, and adolescents” (17, 50). We also assessed the Pediatric Intensity Level of Therapy (PILOT) scale score, a measure of the use of ICP-directed Tier 1 and Tier 2 therapies, for the first 5 days post trauma in surviving patients (51).

**Figure 1:**
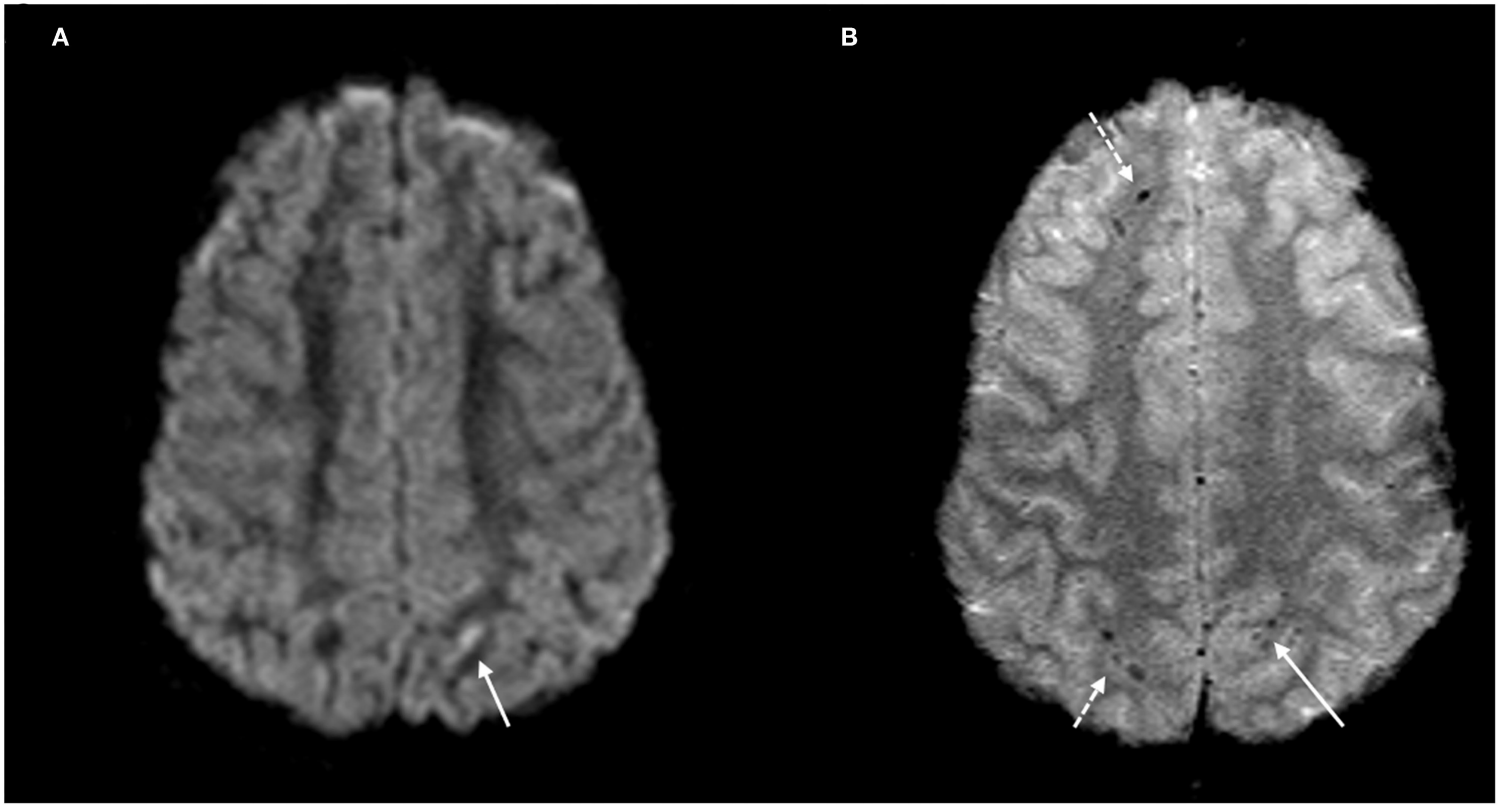
DAI Zone 1 (Superficial) MRI Findings. **(A)** axial diffusion weighted (DWI) and **(B)** axial T2^*^ weighted sequences show a punctate focus of restricted diffusion (**A**, white arrow) at the left parietal gray-white junction with associated susceptibility effect (**B**, solid white arrow). Additional foci of susceptibility within the frontal and parietal subcortical WM (**B**, broken white arrows).

**Figure 2:**
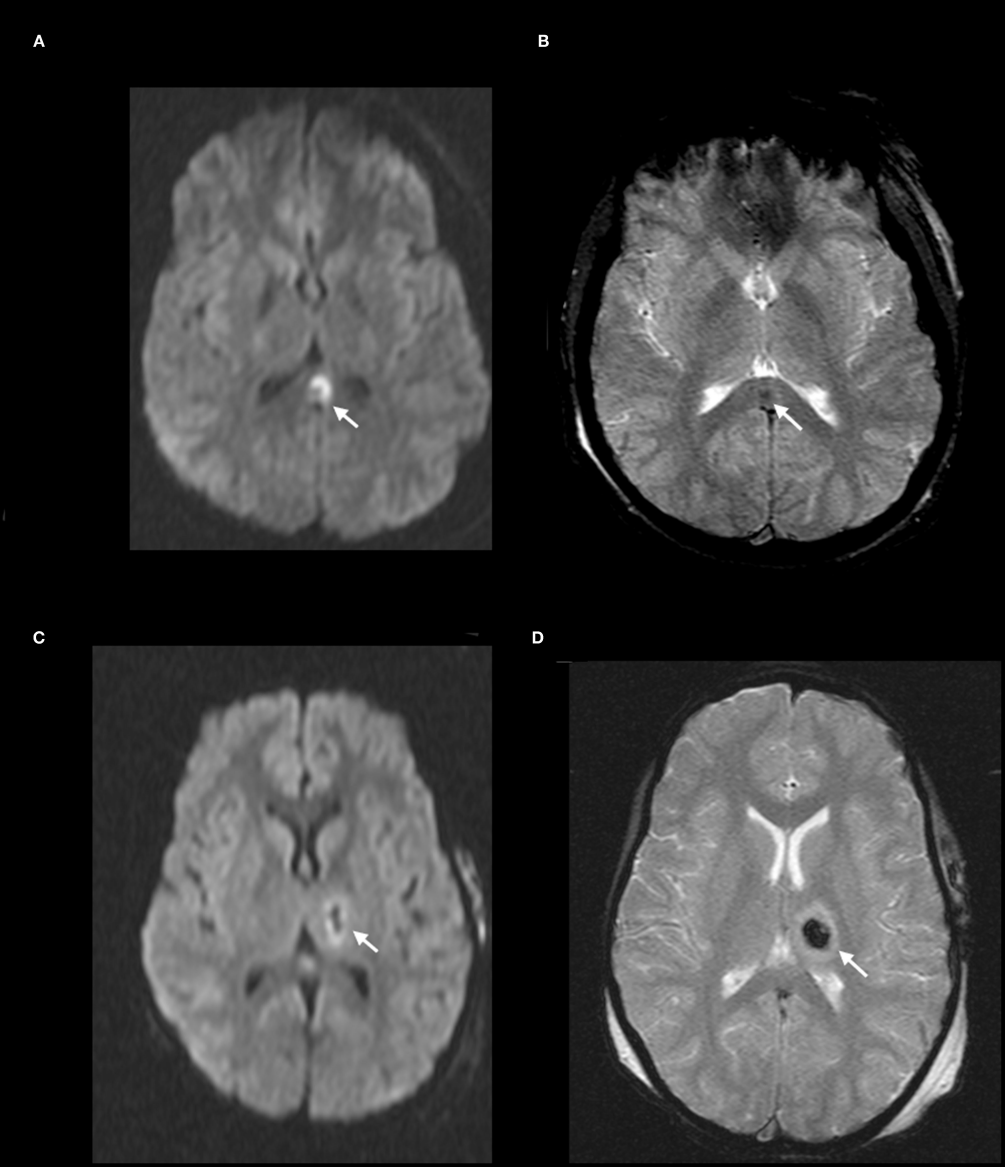
DAI Zone 2 (Deep) MRI Findings. **(A)** axial diffusion weighted (DWI) and **(B)** axial T2^*^ weighted sequences show a focus of restricted diffusion (**A**, white arrow) at the midline splenium of the corpus callosum with associated susceptibility effect (**B**, white arrow). **(C)** axial DWI and **(D)** axial T2^*^ weighted from a different severe pediatric TBI patient with restricted diffusion (**C**, white arrow) and associated susceptibility effect (**D**, white arrow) in the left thalamus.

**Figure 3:**
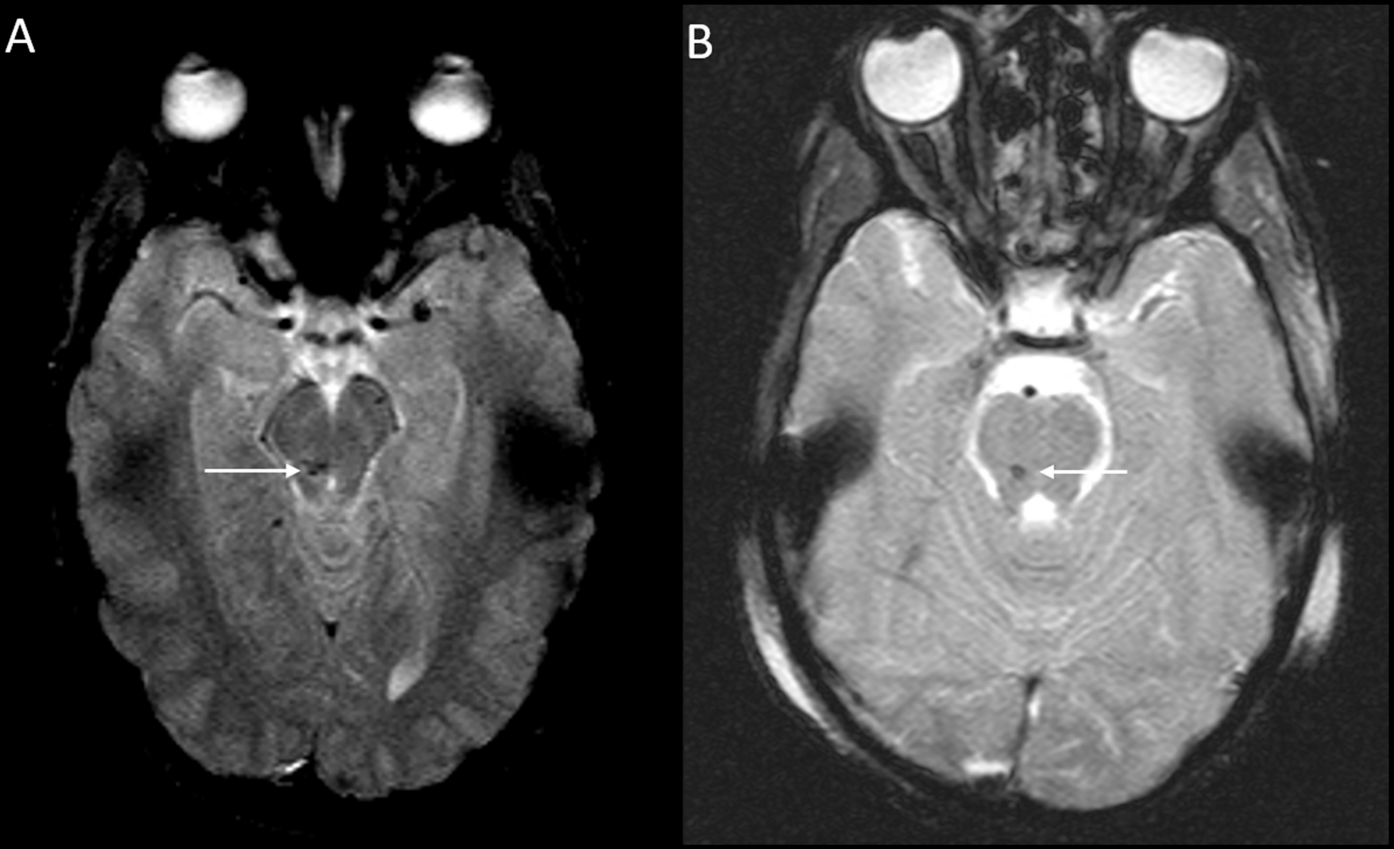
DAI Zone 3 (Posterior fossa) MRI Findings. **(A)** axial T2^*^ weighted image showing susceptibility effect in the right midbrain, and **(B)** axial T2^*^ weighted image in a different severe pediatric TBI patient showing susceptibility effect in the right pons.

When medically stable, MRI was performed within the first week after trauma that confirmed DAI. The MRI sequences included axial and sagittal T1, axial and coronal T2 TSE, axial and coronal fluid-attenuated inversion recovery and axial diffusion-weighted imaging (DWI) on a 1.5 Tesla system. Susceptibility effect was evaluated using either axial T2^*^ weighted gradient-echo (T2^*^) and susceptibility weighted sequences (SWI) sequences. DAI lesions were defined by a board-certified pediatric neuroradiologist (RZ), with >30 years of experience, as hypointense signal on T2^*^ and SWI sequences, and/or restricted diffusion for DWI sequence in white matter structures. No size limit was used. Based on Tong et al. classification of DAI zones in pediatric patients (43), the presence of DAI lesions were qualitatively characterized as being in 1, 2, and/or 3 zones (Figures 1–3):

1 = “superficial” zone- frontal, parietal, temporal, occipital region
2 = “deep” zone- corpus callosum, basal ganglia, thalamus
3 = “posterior fossa” zone- brainstem or cerebellum.

Similar to the established DAI grading system based on depth by Adams et al. (48), this pediatric DAI grading classification is based on increasing depth from zone 1 to zone 3.

### Outcome of Interest

Discharge and long-term Glasgow Outcome Scale-Extended (GOS-E) (up to 10 years following trauma) were obtained on available inpatient and outpatient charts using the pediatric version of the GOS-E (52). In this pediatric GOS-E version:

8 - Death
7 - Vegetative State (VS)
6 - Lower Severe Disability (Lower SD)
5 - Upper Severe Disability (Upper SD)
4 - Lower Moderate Disability (Lower MD)
3 - Upper Moderate Disability (Upper MD)
2 - Lower Good Recovery (Lower GR)
1 - Upper Good Recovery (Upper GR).

Based on previous studies, we defined a “favorable” outcome when GOS-E was 1–4 vs. an “unfavorable” outcome when GOS-E was 5–7 in surviving children, while GOS-E of 8 is death (53, 54). Patients with a GOS-E of 8 (death) were not eligible for follow-up timepoints, and excluded from analyses. None of our patients had withdrawal of life-sustaining therapies.

### Statistical Analysis

#### Association Between Surviving Patient Characteristics and GOS-E on Discharge

Patients' characteristics (age, sex, GCS score, presence of early fever, number of DAI zones, PILOT score) were summarized using median [interquartile ranges, IQR] for numeric variables, and frequency (percent, %) for categorical variables. Because PILOT score was calculated for 5 days, we averaged them for each patient, then reported medians in aggregate. We dichotomized GOS-E as “favorable” outcome (i.e., 1–4) vs. “unfavorable” outcome (i.e., 5–7), and compared patient characteristics by the outcome on discharge. Wilcoxon rank sum test was used for numeric variables, while the Chi-square test or Fisher's exact test was used for categorical variables where appropriate.

#### Association Between Surviving Patient Characteristics and GOS-E Over Time

Frequency (%) of unfavorable outcomes (GOS-E 5–7) was presented at each of the 5 assessment time points (discharge, 6 months, 1, 5, 10 years) for all eligible patients, as well as by patients' characteristics. To assess whether patients with unfavorable outcomes at discharge transitioned to favorable outcomes over time, the proportion of patients with unfavorable outcome at each follow-up time point was compared with the proportion at discharge using the McNemar test. *P*-values were adjusted for multiple comparisons with the Benjamini-Hochberge method. McNemar's test was not conducted for the subgroup of patients based on their characteristics due to the limited sample size. We also compared the differences in proportions of unfavorable outcome between patient groups at each time point using the Fisher's exact test. Multiple comparisons were also adjusted with the Benjamini-Hochberge method.

## Results

Of the 56 children in this study, 2 patients died. The patients were 3 and 4 years old at the time of injury and their initial GCS scores were 3 and 4, respectively. Both had early fever, intracranial hypertension requiring Tier 1 and Tier 2 therapies with median PILOT scores of 22 and 23, respectively, DAI involvement in all 3 zones, deemed non-salvageable for a decompressive craniectomy by the pediatric neurosurgery team and died despite maximal medical life-sustaining therapies.

### Association Between Surviving Patient Characteristics and GOS-E on Discharge

Of the fifty-four children with DAI who survived, median age was 8.5 years [IQR: 5.2, 10.6] at the time of injury (Table 1). At discharge, children with favorable outcome tended to be older, and all of the children who were <5 years old had unfavorable discharge outcome albeit there was a small number of patients (13) in this younger age group. Sex was not associated with discharge outcome. Children who presented with lower GCS (3–5) score on admission were associated with a higher risk of unfavorable discharge outcome. Furthermore, early fever was associated with an unfavorable discharge outcome. More extensive DAI was associated with worse discharge outcome. The median PILOT scores were higher in children with unfavorable outcomes (3.4 [IQR 3.2, 5.7]) compared to those with favorable outcomes at discharge 2.8 [IQR 2.8, 3.2]), *p* < 0.001. We identified 9 surviving patients who needed to receive *all* of the Tier 1 therapy (sedation, analgesia, hyperosmolar therapy and neuromuscular blockade) to control intracranial hypertension and found that the median PILOT scores was significantly higher (7.4 [IQR 6.8, 7.8]) than the majority of the other patients (*n* = 45) who did not need to receive all of the Tier 1 therapy (3.2 [IQR 2.8, 3.4]), *p* < 0.001.

**Table 1:** Patient characteristics by GOS-E at discharge.

		**GOS-E at discharge**		
**Characteristics**	**Overall** ***N* = 54**	**1–4 (Favorable)** ***N* = 24**	**5–7 (Unfavorable)** ***N* = 30**	***p***
**Age (years), median [IQR]**	8.5 [5.2, 10.6]	9.6 [8.0, 13.3]	5.8 [3.9, 9.1]	0.001^*^
**Age group**, ***n*****(%)**				
< 5 years old	13 (24.1)	0 (0.0)	13 (43.3)	<0.001^*^
≥ 5 years old	41 (75.9)	24 (100.0)	17 (56.7)	
**Sex**, ***n*****(%)**				
Female	13 (24.1)	6 (25.0)	7 (23.3)	1.000
Male	41 (75.9)	18 (75.0)	23 (76.7)	
**GCS**, ***n*****(%)**				
3–5	12 (22.2)	2 (8.3)	10 (33.3)	0.046^*^
6–8	42 (77.8)	22 (91.7)	20 (66.7)	
**Early fever**, ***n*****(%)**				
No	35 (64.8)	22 (91.7)	13 (43.3)	<0.001^*^
Yes	19 (35.2)	2 (8.3)	17 (56.7)	
**Number of DAI Zones Involved**, ***n*****(%)**				
1	16 (29.6)	16 (66.7)	0 (0.0)	<0.001^*^
2	19 (35.2)	8 (33.3)	11 (36.7)	
3	19 (35.2)	0 (0.0)	19 (63.3)	
**Average PILOT score, median [IQR]**	3.2 [2.8, 3.4]	2.8 [2.8, 3.2]	3.4 [3.2, 5.7]	<0.001^*^

*PILOT score was assessed for the first 5 days of trauma. For each patient, we computed the average over the 5 days for analysis and presented the median of the average over all patients and by GOS-E at discharge*.

* *p < 0.05 is considered statistically significant*.

### Association Between Surviving Patient Characteristics and Proportion of Unfavorable Outcome (GOS-E 5–7) Over Time

**Figure 4:**
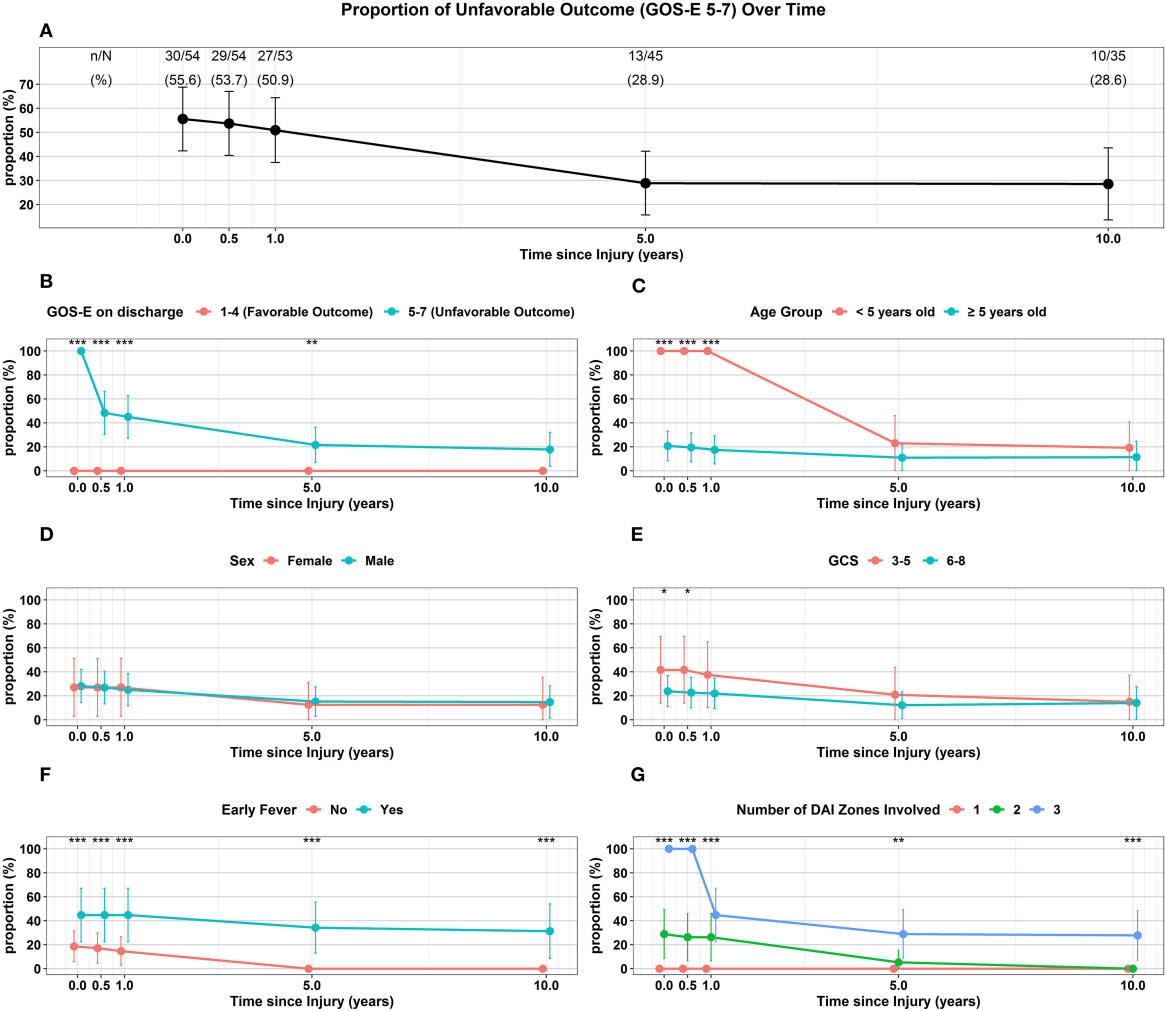
Trajectory of Unfavorable Outcome (GOS-E 5-7) over time with respect to **(A)** Overall proportion of children. **(B)** GOS-E at Discharge. **(C)** Age Group. **(D)** Sex. **(E)** GCS. **(F)** Early Fever. **(G)** Number of DAI Zones Involved. ^***^p < 0.001, ^**^p < 0.01, ^*^p < 0.05 represent differences in proportion of unfavorable outcomes between the groups at the follow-up times.

Of the 54 surviving children, 35 (65%) were followed up to 10 years following injury (Table 2, Figure 4A). Overall, the proportion of unfavorable outcome decreased significantly at follow-up of 5 years (28.9%, 13/45) and 10 years (28.6%, 10/35) from the proportion at discharge (55.6%, 30/54). Of the initial 24 children who had favorable discharge outcome, only 7 children (29%) were followed up to 10 years and all continued to have favorable outcome at 10 years post-injury. Of the initial 30 children who had unfavorable discharge outcome, 28 of the children (93%) were followed up to 10 years with 36% (10/28) continuing to have unfavorable outcome at 10 years post-injury (Table 2, Figure 4B). While the numbers were small, all (13) of the younger children (< 5 years old) demonstrated unfavorable discharge outcome and all were able to be followed up for 10 years with 39% continuing to have unfavorable outcomes at 10 years post-injury. With older children (≥ 5 years old), 22 of the 41 children (54%) were able to be followed up to 10 years and with time, a smaller percentage of children had unfavorable outcome (Table 2, Figure 4C). With regards to sex, 8 of 13 females (62%) and 27 of 41 males (66%) had follow-up for 10 years and with time, both groups had a reduction in unfavorable outcome (Table 2, Figure 4D). With regards to admission GCS, 10 of 12 children (83%) with lower GCS of 3-5 and 25 of 42 children (60%) with higher GCS of 6-8 had follow-up to 10 years. With time both groups of patients had a reduction in proportion of children with an unfavorable outcome (Table 2, Figure 4E).

**Table 2:** Proportion of unfavorable outcome (GOS-E 5–7) over time.

	**Discharge**	**6 months**	**1 year**	**5 years**	**10 years**
**Characteristics**	**30/54 (55.6%)**	**29/54 (53.7%)** ^**+**^	**27/53 (50.9%)** ^**+**^	**13/45 (28.9%)** ^**+**^	**10/35 (28.6%)** ^**+**^
**GOS-E on discharge**					
1-4 (Favorable)	0/24 (0%)	0/24 (0%)	0/23 (0%)	0/15 (0%)	0/7 (0%)
5-7 (Unfavorable)	30/30 (100%)	29/30 (96.7%)	27/30 (90%)	13/30 (43.3%)	10/28 (35.7%)
*p*-value^†^	< 0.001^*^	< 0.001^*^	< 0.001^*^	0.003^*^	0.13
**Age group**					
< 5 years old	13/13 (100%)	13/13 (100%)	13/13 (100%)	6/13 (46.2%)	5/13 (38.5%)
≥ 5 years old	17/41 (41.5%)	16/41 (39%)	14/40 (35%)	7/32 (21.9%)	5/22 (22.7%)
*p* value^†^	< 0.001^*^	< 0.001^*^	< 0.001^*^	0.22	0.55
**Sex**					
Female	7/13 (53.8%)	7/13 (53.8%)	7/13 (53.8%)	3/12 (25%)	2/8 (25%)
Male	23/41 (56.1%)	22/41 (53.7%)	20/40 (50%)	10/33 (30.3%)	8/27 (29.6%)
*p*-value^†^	1	1	1	1	1
**GCS**					
3–5	10/12 (83.3%)	10/12 (83.3%)	9/12 (75%)	5/12 (41.7%)	3/10 (30%)
6–8	20/42 (47.6%)	19/42 (45.2%)	18/41 (43.9%)	8/33 (24.2%)	7/25 (28%)
*p*-value^†^	0.07	0.041^*^	0.14	0.37	1
**Early fever**					
No	13/35 (37.1%)	12/35 (34.3%)	10/34 (29.4%)	0/26 (0%)	0/19 (0%)
Yes	17/19 (89.5%)	17/19 (89.5%)	17/19 (89.5%)	13/19 (68.4%)	10/16 (62.5%)
*p*-value^†^	< 0.001^*^	< 0.001^*^	< 0.001^*^	< 0.001^*^	< 0.001^*^
**Number of DAI Zones Involved**					
1	0/16 (0%)	0/16 (0%)	0/15 (0%)	0/7 (0%)	0/3 (0%)
2	11/19 (57.9%)	10/19 (52.6%)	10/19 (52.6%)	2/19 (10.5%)	0/14 (0%)
3	19/19 (100%)	19/19 (100%)	17/19 (89.5%)	11/19 (57.9%)	10/18 (55.6%)
*p*-value^†^	< 0.001^*^	< 0.001^*^	< 0.001^*^	0.002^*^	0.001^*^

*^+^McNemar's test was used to compare the proportion of Unfavorable outcome (GOS-E 5-7) on follow-ups with the proportion at discharge for all patients. The test was not conducted for each subgroup based on the patient characteristics due to limited sample size*.

† *Fisher's exact test was used to assess the difference in proportion of Unfavorable outcome (GOS-E 5-7) between patient groups at each assessment time*.

*p-values were adjusted using Benjamini-Hochberge Procedure for multiple comparisons*.

* *p < 0.05 is considered statistically significant*.

Of the 35 children that did not have early fever, 19 (54%) of the children were able to be followed up to 10 years while 16 of 19 children (84%) with early fever had follow-up for 10 years; while both groups had a reduction in the proportion of children with unfavorable outcome, none of the children without early fever had unfavorable outcome at 10 years (Table 2, Figure 4F). With regards to the extent of DAI involved, none of the 16 children with only the superficial zone involved on early MRI had unfavorable outcome with only 3 patients (19%) continuing follow-up to 10 years. Of the 19 children with DAI in 2 zones, 11 (58%) of these children had an unfavorable discharge outcome, 14 (74%) of these 19 children had follow-up for 10 years and by this time, none of these children had unfavorable outcome. When all 3 DAI zones were involved, all 19 of the children had unfavorable discharge outcome with 18 or 95% of the children able to be followed up to 10 years; at this 10-year follow-up time, 56% of the children continued to have an unfavorable outcome (Table 2, Figure 4G).

### Characteristics of Patients Lost to Follow-Up

Of the surviving patients, patients lost to follow-up and associations with characteristics are presented in Table 3 (19/54, 35%). The majority of the children lost to follow-up (17/19, 89%) had favorable outcome at discharge while most children (28/35, 80%) who weren't lost to follow-up had unfavorable outcome at discharge. All 19 children that were lost to follow-up were ≥ 5 years old. Sex or admission GCS score were not associated with loss to follow-up. Most children who did not follow-up did not have early fever (16/19, 84%), while 54% (19/35) of children who followed-up did not have early fever. More children (18/19, 95%) who were lost to follow-up had less extensive DAI involved than those who followed-up (9%, 3/35). The median PILOT score was lower in children lost to follow-up than those who were not lost to follow-up.

**Table 3:** Patient characteristics by follow-up status.

		**Lost to follow-up**		
**Characteristics**	**Overall** ***N* = 54**	**Yes** ***N* = 19**	**No** ***N* = 35**	***p***
**GOS-E on discharge**				
1-4 (Favorable)	24 (44.4)	17 (89.5)	7 (20.0)	<0.001^*^
5-7 (Unfavorable)	30 (55.6)	2 (10.5)	28 (80.0)	
**Age (years), median[IQR]**	8.5 [5.2, 10.6]	12.2 [8.8, 15.2]	6.4 [4.0, 8.95]	<0.001^*^
**Age group**, ***n*****(%)**				
< 5 years old	13 (24.1)	0 (0.0)	13 (37.1)	0.002^*^
≥ 5 years old	41 (75.9)	19 (100.0)	22 (62.9)	
**Sex**, ***n*****(%)**				
Female	13 (24.1)	5 (26.3)	8 (22.9)	1.000
Male	41 (75.9)	14 (73.7)	27 (77.1)	
**GCS**, ***n*****(%)**				
3–5	12 (22.2)	2 (10.5)	10 (28.6)	0.178
6–8	42 (77.8)	17 (89.5)	25 (71.4)	
**Early fever**, ***n*****(%)**				
No	35 (64.8)	16 (84.2)	19 (54.3)	0.038^*^
Yes	19 (35.2)	3 (15.8)	16 (45.7)	
**Number of DAI zones involved**, ***n*****(%)**				
1	16 (29.6)	13 (68.4)	3 (8.6)	<0.001^*^
2	19 (35.2)	5 (26.3)	14 (40.0)	
3	19 (35.2)	1 (5.3)	18 (51.4)	
**Average PILOT score, median[IQR]**	3.2 [2.8, 3.4]	2.80 [2.8, 3.2]	3.4 [3.0, 3.4]	0.003^*^

*PILOT score was assessed for the first 5 days of trauma. For each patient, we computed the average over the 5 days for analysis and presented the median of the average over all patients and by follow-up status*.

* *p < 0.05 is considered statistically significant*.

## Discussion

Our overall goal was to describe the long-term trajectory in severe pediatric TBI patients with DAI. The majority of patients survived. Despite a small number of patients in our study, with over 1/3 of the surviving children being lost to follow-up, we were able to describe their long-term outcome up to 10 years. Of the children who had long term follow-up, the proportion of children who had an unfavorable outcome decreased with time. Severely injured children with DAI who had favorable outcome at discharge continued to have favorable outcome up to 10 years. Among the children who had unfavorable outcome at discharge, with long-term follow-up, the majority of these children converted to a favorable outcome. To the best of our knowledge, this study is the first to depict long-term trajectory outcomes of severely-injured children with pure DAI and the first to show an association of fever within the acute post-traumatic period with unfavorable short and long-term outcome.

Previous studies have demonstrated an age-at-injury effect following pediatric TBI with the younger age group having worse outcomes (22–24). In our study, all of the younger children with DAI (< 5 years old) had unfavorable outcome at discharge. Fortunately, the majority of this surviving younger population had a recoverable favorable trajectory. At long term follow-up by 10 years after injury, their proportion of unfavorable outcome was not significantly different than that of older children who were injured. Collectively, our data demonstrated that there was no age-at-injury effect on long-term outcome. Sex had no effect on discharge or long-term outcome on children with traumatic DAI in this study but we recognize that our sample size was small especially with females. Therefore, no firm conclusion about sex effects and long-term outcome on pediatric DAI patients can be drawn and further studies with a larger number of patients, especially the female population, need to be pursued.

Lower admission GCS score has been associated with worse outcome in pediatric and adult patients with DAI (43, 55). While a lower admission GCS was associated with an unfavorable outcome at discharge, there was no difference in long-term outcome compared to those with a higher admission GCS in our study. However, our sample size was small and our dichotomized GCS scale comparisons were all within the “severe” GCS range while Skandsen and Tong's group compared GCS from mild, moderate and severe GCS ranges.

To the best of our knowledge, this is the first study to show an association of fever within the acute post-traumatic period with both worse short-term and long-term outcome in severe pediatric TBI patients with DAI. In a previous pediatric TBI study, 30% of children had early fever on the first day after injury and severe initial injury (GCS ≤ 8) or DAI as the pathology were risk factors that predicted early fever (20). Another pediatric study demonstrated 29% of their children had early fever within the 1st day following severe pediatric TBI (21). Both of these studies demonstrated that the presence of fever was associated with worse outcome at discharge but no long-term outcome assessments were done. In our current study, none of the children with early fever had an obvious infectious source- as blood, urine and sputum cultures were negative for an infection and none of our children were treated with a ventriculostomy. The etiology of non-infectious fever following TBI is thought to be multifactorial and has been attributed to neuronal excitotoxicity, the inflammatory response, disruption of the blood-brain barrier, intraparenchymal blood, catecholamine release, and alteration of the hypothalamic thermoregulatory center (56–58). While further mechanistic studies need to be done, perhaps a diffuse brain lesion due to traumatic DAI puts these patients at more risk for the multifactorial hyperthermia response. Furthermore, our study suggests the importance of early targeted temperature management (TTM) in pediatric TBI (59).

All of our children with only the superficial DAI zone involved all had favorable recovery. In children with the superficial and deep zones involved but not the posterior fossa, the majority of the patients had unfavorable discharge outcome but eventually recovered with favorable outcome at long-term follow-up at 10 years. It is not surprising that involvement in all 3 DAI zones (superficial, deep and posterior fossa) within the first week following injury was associated with the worst discharge outcome. With long-term follow-up, while some of these children were fortunately able to recover, however, the majority still had unfavorable outcome. The fact that involvement of deeper brain lesions following pediatric TBI may be associated with worse outcome is consistent with the Ommaya-Generalli hypothesis (60).

While early pathophysiologic events such as intracranial hypertension is a risk factor for poor outcome following severe TBI in children (17–19), the role of ICP in children with traumatic DAI has not been extensively studied. In the landmark study by Adams et al. (61), 25 out of 45 patients (56%) with DAI had pathologic concerns for elevated ICP compared to 114 out of 132 patients with non-DAI (86%) (61); so while the pathologic concern for elevated ICP was significantly less in the DAI patients than in the non-DAI group, it appears that intracranial hypertension may not be a rare event in the DAI population. In another study, the prevalence of intracranial hypertension episodes was low with 6% of the patients requiring treatment for intracranial hypertension (62). In other studies, intracranial hypertension was more prevalent between 33 and 58% of the adult patients and was associated with more extensive DAI, including DAI in the posterior fossa and was associated with worse short-term outcome (63, 64). In one pediatric study with traumatic DAI, 81% of the ICP-monitored patients had an “episode” of intracranial hypertension which was more prevalent in children with more extensive DAI especially in the superficial zone. There was no effect on short term outcome (43). In our study, the overall PILOT scores were low, demonstrating that most of our surviving DAI children did not have a profound ICP-directed therapy burden due to a low prevalence of intracranial hypertension. Only a very small minority of surviving children in our study needed all of the Tier 1 therapy for intracranial hypertension resulting in a higher PILOT score. Overall with our limited sample size, no assessments on ICP and its effect on long-term outcome can be assessed. Future studies with a much more robust population of patients or analysis of the large ADAPT database (65) should be done on the role of ICP monitoring, the prevalence of intracranial hypertension and ICP-directed therapies and its effect on outcome in children with traumatic DAI.

### Study Limitations

As previously mentioned, one major limitation of this study was that there was a small number of patients with over 1/3 of the patients lost during the 10 years of follow-up. As already described in Table 3, the majority of the children who were lost to follow-up had favorable outcome at discharge. Also, three guidelines related to severe pediatric TBI, many changes in PICU care and TBI outcomes have occurred during the 17 years of this study which may have hampered our analysis of clinical factors associated with long-term outcome. Another major limitation to our study is the exclusion of pediatric TBI patients with abusive head trauma, which is one of the leading causes of severe TBI in the youngest population given that our study had a small number of patients who were < 5 years of age. We also excluded patients with other co-morbidities, such as polytrauma patients or children with any past medical history which further limits our data interpretation. None of the pediatric patients in this study were treated with additional neurosurgical procedures (such as a decompressive craniectomy) besides an ICP monitor. While the goal of this study was to only examine a pure pediatric DAI group, future studies should address the contribution of abusive head trauma, polytrauma, those that needed a decompressive craniectomy and other co-morbidities and its effect on the trajectory of long-term outcome to characterize the pediatric DAI population more completely.

Another limitation is that one of our inclusion criteria was that the admission CT “concerning for DAI with microhemorrhages in the white matter tracts without a focal mass lesion” may bias the sample toward the most severe TBI patients and will miss patients with non-hemorrhagic DAI. Our intent in this study was to describe severe pediatric TBI patients with DAI but not mild-moderate severity patients with DAI. Another limitation is that the MRI was performed early (within the first week) following trauma which may have underestimated the extent of long-term imaging sequelae of traumatic DAI, which may evolve with time following injury. MRI interpretation for this study was a qualitative analysis to simply identify the presence or absence and location of DAI lesions. This was performed by a board-certified pediatric neuroradiologist who was blinded to the patient's clinical pathophysiologic course and outcome, but was aware of the diagnosis of TBI. Other pediatric TBI studies have demonstrated the utility of qualitative MRI analysis (31, 66, 67), which is commonly used in radiology practice and lends itself to the clinical interpretation of images. These MRI images were performed on a 1.5 Tesla scanner over 10 years ago and advanced neuroimaging methods have rapidly improved over the long time frame of this study. Since our MRI protocols changed over the 17 years of patient follow-up, many subjects were imaged using a T2^*^ gradient-weighted sequence, which has decreased sensitivity for hemorrhage, when compared with the current SWI sequences. Additionally, imaging sequences such as diffusion tensor imaging (DTI) may be useful for investigating white matter integrity in TBI, but were not routinely available during this study (68). However, this study was not intended to validate imaging diagnostic criteria for the evaluation of patients with DAI; rather, the emphasis of this work was toward assessing the clinical follow-up of patients who previously met the diagnostic criteria for DAI. This extensive follow-up period affords an important perspective on the relationship between diagnosis and the long-term clinical outcomes of patients.

We used the pediatric version of GOS-E as our outcome assessment. While GOS-E remains one of the most common outcome measures used in TBI studies, we acknowledge that it is a very global outcome measure and cannot answer more specific granular data such as cognition, memory, and psychosocial function.

## Conclusion

We describe the long-term trajectory of the outcome of severe pediatric TBI patients with pure DAI. While this was a single institution study with a small sample size, and over one-third of surviving children were lost to follow-up, the majority of the children survived. For the surviving children who had follow-up for 10 years after injury, the majority of these children made recovery to favorable outcome. Further studies are needed to better understand the pathophysiology of traumatic DAI in children to optimize their acute and long-term care with the ultimate hopes of improving outcome.

## Data Availability Statement

The original contributions presented in the study are included in the article/supplementary material, further inquiries can be directed to the corresponding author/s.

## Ethics Statement

The studies involving human participants were reviewed and approved by the Committee for the Protection of Human Subjects of the Children's Hospital of Philadelphia Research Institute Internal Review Board- IRB 16-013395. Written informed consent from the patients or patients legal guardian/next of kin was not required to participate in this study in accordance with the institutional requirements.

## Author Contributions

S-SL was the primary author of this paper and was involved with data analysis, review of the literature, and critically writing and revising the paper. TK, SF, and CK were involved with data collection, outcome data collection, and revising the manuscript. PS, GH, VM, AT, and RR were involved in writing and revising the manuscript. SS was involved with MRI interpretations, figure selection, and critically writing and revision the manuscript. BZ, SA, and HG were the statisticians involved in analyzing the data and critically writing and revising the manuscript. JH was the senior author on this manuscript and was involved in conception, data collection, data analysis, and critically writing and revising the paper. All contributing authors have reviewed and approved the manuscript for submission with adherence to ethical standards.

## Conflict of Interest

The authors declare that the research was conducted in the absence of any commercial or financial relationships that could be construed as a potential conflict of interest.

## Publisher's Note

All claims expressed in this article are solely those of the authors and do not necessarily represent those of their affiliated organizations, or those of the publisher, the editors and the reviewers. Any product that may be evaluated in this article, or claim that may be made by its manufacturer, is not guaranteed or endorsed by the publisher.
